# Imaging biomarkers for steatohepatitis and fibrosis detection in non-alcoholic fatty liver disease

**DOI:** 10.1038/srep31421

**Published:** 2016-08-12

**Authors:** Rocío Gallego-Durán, Pablo Cerro-Salido, Emilio Gomez-Gonzalez, María Jesús Pareja, Javier Ampuero, María Carmen Rico, Rafael Aznar, Eduardo Vilar-Gomez, Elisabetta Bugianesi, Javier Crespo, Francisco José González-Sánchez, Reyes Aparcero, Inmaculada Moreno, Susana Soto, María Teresa Arias-Loste, Javier Abad, Isidora Ranchal, Raúl Jesús Andrade, Jose Luis Calleja, Miguel Pastrana, Oreste Lo Iacono, Manuel Romero-Gómez

**Affiliations:** 1 UCM Digestive Diseases and CIBEREHD, Virgen Macarena-Virgen del Rocío University Hospitals, University of Sevilla, Sevilla, 41013, Spain; 2 Instituto de Biomedicina de Sevilla (IBiS), University of Sevilla, Sevilla, 41013, Spain; 3 Group of Interdisciplinary Physics, Engineering School, University of Sevilla, Sevilla, 41092, Spain; 4 Pathology Unit, Valme University Hospital, University of Sevilla, Sevilla, 41014, Spain; 5 Radiology Unit, Valme University Hospital, University of Sevilla, Sevilla, 41014, Spain; 6 Medical Sciences, University of Torino, Torino, 10126, Italy; 7 IDIVAL, Marqués de Valdecilla University Hospital, Gastroenterology and Hepatology Service, Santander, Spain; 8 Radiology Unit, Marqués de Valdecilla University Hospital, Santander, 39008, Spain; 9 UCM Digestive Diseases, Valme University Hospital, Sevilla, 41014, Spain; 10 UCM Digestive Diseases and CIBEREHD, Virgen de la Victoria University Hospital, Málaga, 29010, Spain; 11 UCM Digestive Diseases, Tajo University Hospital, Madrid, 28300, Spain; 12 UCM Digestive Diseases, Puerta de Hierro University Hospital, Madrid, 28222, Spain; 13 Radiology Unit, Puerta de Hierro University Hospital, Madrid, 28222, Spain

## Abstract

There is a need, in NAFLD management, to develop non-invasive methods to detect steatohepatitis (NASH) and to predict advanced fibrosis stages. We evaluated a tool based on optical analysis of liver magnetic resonance images (MRI) as biomarkers for NASH and fibrosis detection by investigating patients with biopsy-proven NAFLD who underwent magnetic resonance (MR) protocols using 1.5T General Electric (GE) or Philips devices. Two imaging biomarkers (NASHMRI and FibroMRI) were developed, standardised and validated using area under the receiver operating characteristic curve (AUROC) analysis. The results indicated NASHMRI diagnostic accuracy for steatohepatitis detection was 0.83 (95% CI: 0.73–0.93) and FibroMRI diagnostic accuracy for significant fibrosis determination was 0.85 (95% CI: 0.77–0.94). These findings were independent of the MR system used. We conclude that optical analysis of MRI has high potential to define non-invasive imaging biomarkers for the detection of steatohepatitis (NASHMRI) and the prediction of significant fibrosis (FibroMRI) in NAFLD patients.

Non-alcoholic fatty liver disease (NAFLD) is commonly diagnosed when evidence of steatosis, obtained either by imaging or histology, is found in the absence of significant alcohol consumption, viral infection, and autoimmune or drug-related liver injury^1^. About a third of the overall population currently suffer from any stage of NAFLD^2^. NAFLD is a clinico-pathological entity that ranges from hepatic fat accumulation (simple steatosis) to non-alcoholic steatohepatitis (NASH), which is a progressive form that may lead to fibrosis^3^, cirrhosis and ultimately hepatocellular carcinoma^4^,^5^. Furthermore, liver fibrosis is the strongest predictor to long-term overall mortality and liver-related events^6^. Patients with NAFLD almost invariably display insulin resistance, together with other morbid-mortality risk factors such as overweight, visceral adiposity, diabetes, hyperlipidaemia and high blood pressure. These patients also show an augmented rate of mortality compared to general population paired by age and sex^7^.

Unmet needs in NAFLD management include: a) NASH detection that could help exclude patients not at risk of disease progression; b) Prediction of significant fibrosis to select patients with poorer prognosis and survival^8^. Indeed, the strongest predictor of fibrosis progression in NAFLD is the presence of steatohepatitis^9^. Percutaneous liver biopsy remains the gold standard for diagnosis of steatohepatitis and fibrosis staging^10^. However, beyond the well-documented limitations such as high costs, morbidity and sampling error in evaluating steatohepatitis, the intra- and inter-observer variability makes the diagnosis very difficult^11^,^12^. Hence, the development of a definitive non-invasive test would be desirable. Several quantitative scores such as the NAS (NAFLD Activity Score) and SAF (Steatosis, Activity, Fibrosis) Score have been developed, but defining NASH from the quantitative score is neither easy nor accurate^13^,^14^. Several imaging tests have emerged to help diagnosis. These include transient elastography^15^, acoustic radiation force impulse^16^, and magnetic resonance elastography^17^. More recently, serum biomarkers have been demonstrated to be moderately useful. These include cytokeratin-18 (CK-18), fibroblast grown factor 21 (FGF21)^18^,^19^ and NAFLD fibrosis score, in combination with non-invasive methods developed in the hepatitis C setting e.g. the Sydney Index. Optical analyses of liver images have demonstrated usefulness in fibrosis prediction related to hepatitis C^20^. Additionally, magnetic resonance methods are highly specific and appraise the entire organ, becoming an attractive alternative to invasive procedures.

In the current study, the main aim was to develop, standardise and validate imaging biomarkers defined by optical processing methods applied to conventional non-enhanced contrast magnetic resonance images (MRI) in order to predict, using non-invasive tools, steatohepatitis and fibrosis stages in NAFLD patients. The secondary objective was to compare these imaging biomarkers with currently available non-invasive markers.

## Results

### Development and standardisation of NASHMRI to detect steatohepatitis

Estimator E3 (harmonic mean) from MRI protocol SSFSE-T2, estimator E57 (second order contrast) from DYNAMIC MRI protocol, and estimator E73 (averaged mean curvature) from MRI protocol FAST-STIR, were found to be independently associated with NASH. Model coefficients associated with each one of these independent variables were β_1_ = 0.079 (OR: 1.08, 95% CI: 1.02–1.15; p = 0.015) and β_2_ = 0.127 (OR: 1.14, 95% CI: 1.03–1.26; p = 0.015). The influence of these estimators on the predictive equation to obtain the probability of suffering steatohepatitis was developed on estimation cohort and is given by:


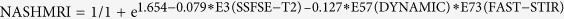


In the estimation cohort (n = 39), AUROC obtained was 0.88 (95% CI: 0.77–0.99). Mean NASHMRI discriminated between simple steatosis and steatohepatitis, with high sensitivity (Se) and specificity (Sp). The best cut-off (based on Se and Sp) to segregate patients according to steatohepatitis presence or absence was 0.5; patients with a NASHMRI score > 0.5 were considered as NASH. With this threshold, Se was 87%, Sp 74%, positive predictive value (PPV) 80% and negative predictive value (NPV) 82%.

In the validation cohort (n = 87), NASHMRI AUROC obtained was 0.83 (95% CI: 0.75–0.92). Using the defined threshold of 0.5 for NASHMRI prediction, the results achieved were: Se 87%, Sp 60%, PPV 71% and NPV 81%.

### Definition of FibroMRI for significant fibrosis prediction

Estimator E22 (Pearson’s asymmetry coefficient) from MRI protocol SSFSE-T2 and estimators E3 (harmonic mean), E6 (mode), E31 (column’s mean of multi-oriented co-occurrence matrix) and E75 (maximum of main curvatures) from MRI protocol DYNAMIC were found to be independently associated with fibrosis. Model coefficients associated with each of these independent variables were: β_1_ = 1.101 (OR: 3.01, 95% CI: 1.25–7.25; p = 0.014); β_2_ = −1.105 (OR: 0.33, 95% CI: 0.14–0.77; p = 0.010); β_3_ = −115.737 (OR: 0.08, 95% CI: 0.02–0.14; p = 0.046); β_4_ = 0.696 (OR: 2.00, 95% CI: 1.19–3.38; p = 0.009); and β_5_ = −0.825 (OR: 0.44, 95% CI: 0.21–0.93; p = 0.030). Their introduction into the predictive equation defining the risk of suffering significant fibrosis was:


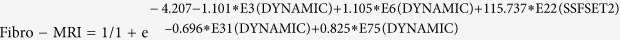


In the estimation cohort (n = 39), AUROC obtained was 0.94 (95% CI: 0.87–1.00). FibroMRI differentiated between mild (F0-F1) and significant (F2-F3-F4) fibrosis, without any overlap. The best cut-off, determined by Se and Sp, to segregate patients with absence or presence of significant fibrosis was 0.5; those patients with a FibroMRI >0.5 were considered as suffering from significant fibrosis (See Supplementary material). With the previously defined cut-off point of 0.5 for FibroMRI, the results obtained were: Se 81%, Sp 85%, PPV 77% and NPV 86%. In the validation cohort (n = 87), FibroMRI AUROC for significant fibrosis was 0.85 (95% CI: 0.77–0.93). With the defined threshold at 0.5 for FibroMRI prediction, the results obtained were: Se 77%, Sp 80%, PPV 67% and NPV 87%.

Number of patients suffering from advanced fibrosis and/or cirrhosis was not enough to define outright thresholds beyond significant fibrosis. Nevertheless, FibroMRI correlated with fibrosis stage (r = 0.54; p < 0.0001), independently of the device used (GE r = 0.54; p < 0.001 and Philips r = 0.44; p < 0.002). Finally, FibroMRI was found different according to the stage of fibrosis: F0 (n = 36) 0.16 ± 0.24 [95% CI 0.07–0.24]; F1 (n = 16) 0.34 ± 0.40 [95% CI 0.12–0.55] and F > 2 (n = 30) 0.64 ± 0.30 [95% CI 0.53–0.75]; p < 0.0001.

### Comparative analyses of NASHMRI and FibroMRI

#### Standardisation of NASHMRI and FibroMRI across MRI systems

NASHMRI calculated using GE scanners (n = 35) showed a similar diagnostic accuracy when compared with NASHMRI calculated in patients who underwent MRI using the Philips system (n = 52) i.e. AUROC = 0.75 (95% CI: 0.56–0.95) *vs.* AUROC = 0.85 (95% CI: 0.73–0.97), respectively (p = ns). Regarding FibroMRI, evaluations performed using GE MRI scanners showed an AUROC of 0.80 (95% CI: 0.65–0.95) *vs.* AUROC of 0.84 (95% CI: 0.72–0.96) using the Philips system (p = ns). Scores yielded by both scanners are comparable, and the same thresholds for NASHMRI and FibroMRI applied to both devices (GE or Philips). Spearman coefficient together with diagnostic accuracy was similar for both scanners and end-points.

Both machines pose the same image quality and resolution, and were processed likewise with FibroMRI and NASHMRI without distinctions. In a subset of 9 patients both studies were available (6 w/o fibrosis and 5 w/o NASH). Fibrosis was detected by both methods in 3/3 cases and excluded fibrosis in 5/6 cases without this condition using both Philips and GE devices. Besides, NASH was confirmed in 3/4 cases by both techniques and excluded in 4/5 cases. Further analysis including a large cohort of patients would better define the reproducibility of these results.

### Comparative analysis with non-invasive biochemical markers of steatohepatitis

**Figure 1:**
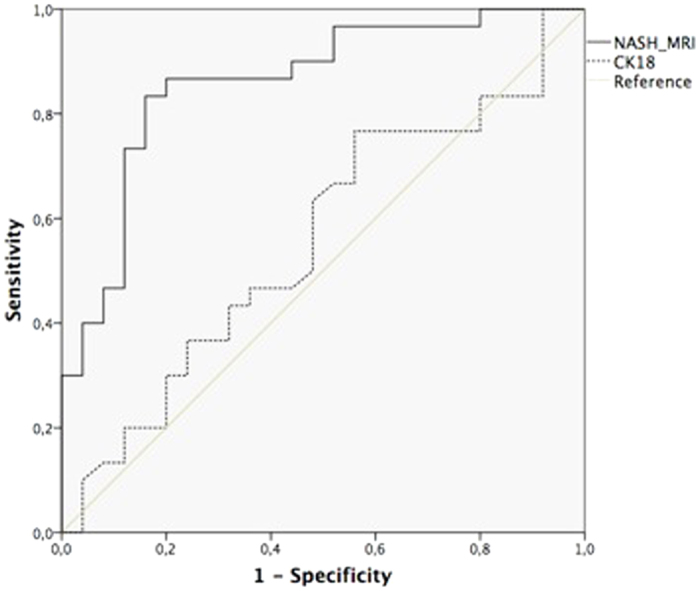
Analysis of diagnostic accuracy for NASH detection comparing NASHMRI and CK-18 (AUROC: 0.86; 95% CI: 0.76–0.96 *vs.* AUROC: 0.44; 95% CI: 0.29–0.60, respectively; p < 0.0001).

NASHMRI was compared with CK-18 levels in NASH diagnosis. NASHMRI offered the best diagnostic accuracy with an AUROC of 0.86 (95% CI: 0.76–0.96) for steatohepatitis presence. This was significantly better than CK-18 levels, which showed an AUROC of 0.56 (95% CI: 0.40–0.71; p < 0.05) (Fig. 1). NAS score correlated significantly with NASHMRI (r = 0.38; p < 0.001) and CK-18 levels (r = 0.29; p < 0.02).

### Comparative analysis with non-invasive biochemical markers of significant fibrosis

**Figure 2:**
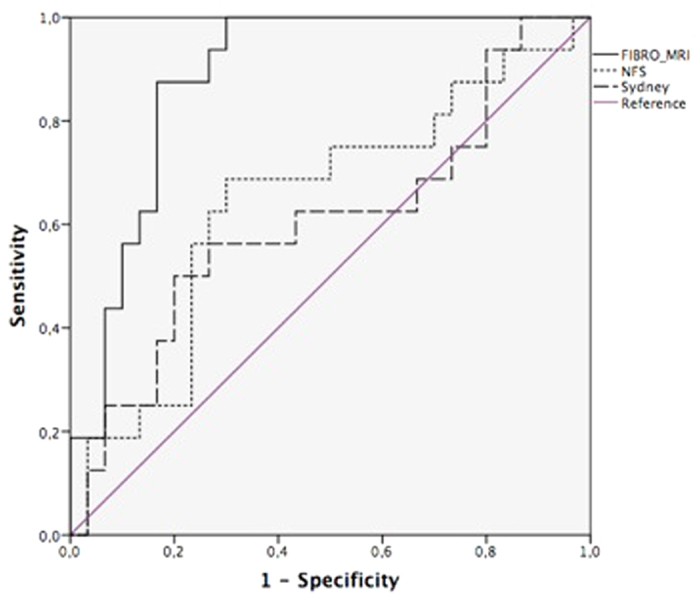
Analysis of diagnostic accuracy for significant fibrosis (≥F2) comparing FibroMRI, Sydney Index and NAFLD Fibrosis Score (AUROC: 0.85; 95% CI: 0.74–0.97 *vs.* AUROC: 0.69; 95% CI: 0.50–0.87 *vs.* AUROC: 0.76; 95% CI: 0.61–0.91, respectively; p < 0.001).

**Figure 3:**
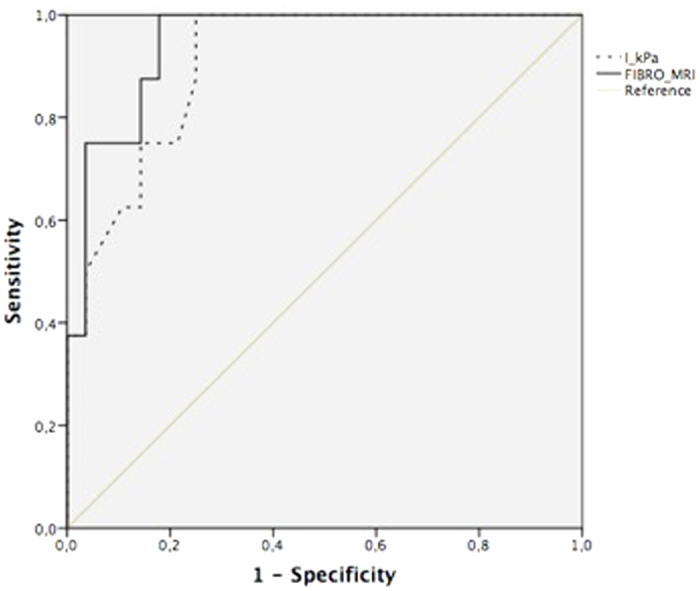
Analysis of diagnostic accuracy for significant fibrosis (≥F2) detection comparing Fibro-MRI and valid transient elastography measurements (AUROC: 0.95; 95% CI: 0.88–1.00 *vs.* AUROC: 0.91; 95% CI: 0.81–1.00, respectively; p = ns).

FibroMRI was significantly superior to NFS and Sydney Index (AUROC: 0.85; 95% CI: 0.74–0.97 *vs.* AUROC: 0.76; 95% CI: 0.61–0.91 *vs.* AUROC: 0.69; 95% CI: 0.50–0.87, respectively; p < 0.05) (Fig. 2) in predicting significant fibrosis. Fibrosis stage correlated with FibroMRI (r = 0.61; p < 0.001), and NFS (0.52; p < 0.001). Also, a significant correlation between NFS and FibroMRI was observed (r = 0.53; p < 0.001). Lastly, findings with FibroMRI were similar to that of transient elastography (AUROC: 0.95; 95% CI: 0.88–1.00 *vs.* AUROC: 0.91; 95% CI: 0.81–1.00, respectively; p = ns) (Fig. 3).

## Discussion

Computerised optical analysis of conventional non-contrast-enhanced MR images of the liver enables the detection of steatohepatitis by NASHMRI and significant fibrosis by FibroMRI in patients suffering from NAFLD. This study addresses an important need for non-invasive markers of both NASH and the associated fibrosis. Since fibrosis and steatohepatitis generate appreciable architectural changes in liver structure, it would be possible, using this software, to forecast the rate of disease progression, to support therapeutic decision-making, and to monitor potential effects of therapy. Diagnoses of liver diseases have long relied on liver biopsy, despite their high intra- and inter-observer variability, discomfort to the patient, and sampling error^21^. A panel of serum biomarkers to confirm, or rule out, steatohepatitis has remained elusive. The NashTest, included in FibroMax^22^, is a semi-quantitative score with a wide grey zone, OWLiver^23^ accurately predicts steatohepatitis. However, it needs to be analysed in a centralised laboratory, and which undermines its accessibility^24^. Lastly, CK-18^25^ failed to confirm any usefulness in the diagnosis of NASH^26^. Non-invasive diagnosis of significant fibrosis in NAFLD is also a challenge. NAFLD Fibrosis Score^20^ was specifically developed for NAFLD, however, it showed a wide grey zone in the validation process^27^. Non-invasive markers shunted from hepatitis C evaluations have been tested in NAFLD. These include Sydney, FIB-4, Forns and APRI indexes. However, poor correlations between serum biomarkers of liver fibrosis (APRI, FIB-4, AST/ALT ratio, European Liver Panel and Liver stiffness measurement) were reported in diabetic patients. Agreement was good with respect to absence of advanced liver disease, but not in patients with progressive disorders^28^. FibroMRI was superior to NFS and Sydney Index in predicting significant fibrosis. These results would be expected because NFS was designed to predict advanced fibrosis from significant fibrosis, and Sydney Index was developed in patients with chronic hepatitis C.

Currently, image-based non-invasive methods are receiving increasing attention. Ultrasonography, transient elastography, acoustic radiation force impulse, magnetic resonance spectroscopy, magnetic resonance elastography have been employed for NASH diagnosis. Ultrasonography has shown 60–94% sensitivity and 84–95% specificity in hepatic steatosis detection^29^; an acceptable first-line steatosis-screening tool in clinical practice^30^ but which cannot distinguish NASH from simple steatosis^31^. Transient elastography (FibroScan; Echosens, Paris, France)^32^ has shown a respectable diagnostic accuracy in stratifying advanced fibrosis in NAFLD^33^. However, transient elastography was found not to be useful in NASH diagnosis^34^ since >10% of patients could not be assessed because of procedure failures due, mainly, to high body mass index (BMI)^35^. Hence, thresholds to define advanced fibrosis stages remain controversial. Higher scores of stiffness (kPa) to define cirrhosis are required compared to cut-offs accepted for viral hepatitis^36^,^37^. Magnetic resonance spectroscopy (MRS) enables the evaluation, *in vivo,* of liver molecular composition, and detects steatosis with high accuracy^38^. It is the reference method for steatosis, but fails in NASH detection. Magnetic resonance elastography (MRE) has been shown to be accurate in fibrosis staging^39^,^40^ but its availability is low in most centres and needs further external validation. The main limitation of these image-based methods remains their inability to detect steatohepatitis. Novel developments in the MR field, such as gadolinium probes targeted to type-1 collagen, have shown excellent preliminary results but still need to be translated into the standard clinical setting^41^. The scores generated are related to the presence of steatohepatitis or fibrosis; the lower the scores the lower the probability of suffering from steatohepatitis or significant fibrosis. The opposite is also valid i.e. the higher the score the greater the risk of displaying steatohepatitis or significant fibrosis. Studies comparing different MRI systems manufacturers (such as Siemens vs. Phillips systems) are warranted.

FibroMRI accurately predicts significant fibrosis stages. MRI can access deep tissue fibrosis staging while analysing the whole liver, and saving on sampling errors. As such, it could be useful in the management of liver donors prior to liver transplantation^42^,^43^. Also, it could be tested in liver diseases that share steatosis as a major feature such as, for example, viral hepatitis or alcohol-related liver diseases. Since ionizing radiation is avoided, this technique would be suitable for harmlessly monitoring fibrosis and steatohepatitis progression over time. Progression from simple steatosis to NASH and fibrosis in paired liver biopsies 3 to 6 years apart has been reported recently^44^,^45^. Further, in 51 patients who had undergone two liver biopsies and scored separately, the results indicated that steatosis, ballooning, inflammation and fibrosis appeared not to be equally distributed across the liver. In 21 of 51 cases there was one stage difference in the degree of fibrosis, while ballooning was detected in only one of the liver biopsies in 9 of 51 cases^19^. As such, close follow-up of the progression of liver disease in NAFLD is mandatory, making non-invasive imaging biomarkers the optimal approach.

The main technical limitation of this technique is segmentation error, because the method is based on an optical analysis of images to quantify differences not perceptible to the naked eye. Presence of vessels or different structures in the sample studied could be confounding factors resulting in under- or over-estimation of the degree of fibrosis, or inflammation. This problem can be solved, as in the current analysis, by excluding areas containing blood vessels, biliary tract, or focal lesions and, as well, all samples with >30% pixels outside the segmented area. To avoid manual segmentation errors, this process has been automated, thus allowing the translation of the study to different liver diseases. External validation studies are warranted.

Among the main strengths of the study is the demonstration of its applicability at different sites, and using two types of MR devices. The parameters derived from both types of machines are standardised. Further, study design was such as to minimise observer-related variation, including different measurement conditions that could impinge on diagnostic accuracy.

In conclusion, NASHMRI and FibroMRI could be useful in diagnosing steatohepatitis and significant fibrosis in patients with suspected NAFLD. These imaging biomarkers offer clear advantages above liver biopsy since they are innocuous, less traumatic for the patient, and cheaper. Analysing the whole liver using user-friendly software would be ideal for close monitoring over time, and for wide implementation for screening large numbers of at-risk patients. Clear disease staging with respect to severity would provide support in clinical decision-making.

## Methods

### Study design and patients

This was a cross-sectional and multi-centred study that included 126 well-characterised biopsy-proven NAFLD patients who were recruited between June 2009 and June 2013. Estimation cohort was enrolled from June 2009 to September 2010, and validation set from January 2010 to June 2013. Clinical data were collected at the time of liver biopsy using a special case record form, together with blood samples for biochemical analyses. The study protocol conformed to the ethical guidelines of the 1975 Declaration of Helsinki. The Institutional Review Board Committee from each participating hospital approved the study protocol (Virgen Macarena-Virgen del Rocío University Hospitals, Valme University Hospital, Città della Salute e della Scienza di Torino Hospital, Marqués de Valdecilla University Hospital, Virgen de la Victoria University Hospital, Tajo University Hospital and Puerta de Hierro University Hospital). All patients provided informed consent for liver biopsy, MRI study and blood extraction. All data were coded to ensure anonymity. Exclusion criteria were: significant alcohol abuse (>30 g/day in men and >20 g/day in women); evidence of viral or autoimmune hepatitis or HIV; drug-induced fatty liver; other metabolic liver diseases (such as haemochromatosis or Wilson’s disease); pregnancy; parenteral nutrition. The study sample was composed of all patients who fulfilled the inclusion criteria and were not disqualified by one or more of the exclusion criteria. Untreated and histologically-confirmed NAFLD patients were recruited as part of the FLIP (Fatty Liver: Inhibition of Progression; www.flip-fp7.eu) project. Patients enrolled in the study were classified according to sex, age, fibrosis stage and presence/absence of steatohepatitis (Table 1). Patients underwent a complete medical history, physical examination, liver biopsy and imaging study. An overnight (12 h) fasting blood sample was taken at the same time of liver biopsy for routine biochemical analyses that included the transaminases (ALT, AST), alkaline phosphatase, γGT, total cholesterol and triglycerides. Fasting samples of serum obtained after centrifugation were stored in aliquots at −80 °C until assayed. Serum insulin levels were measured by electrochemiluminescence immunoassay, using an Elecsys 1010/2010 autoanalyzer (Elecsys MODULAR ANALYTICS E170; Roche, Basil, Switzerland). CK-18 was measured using a human ELISA Kit (Abnova, Walnut, CA, USA). NAFLD Fibrosis Score^46^ and Sydney Index^47^ were calculated as previously reported. Transient elastography was measured using FibroScan (Echosens, France). Height and weight were determined at baseline and, from which, the body mass index (BMI) was calculated as weight (in kg) ÷ height (in m^2^).

### Histological staging and grading

Percutaneous liver biopsies were performed under local anaesthesia and ultrasound guidance. Liver specimens were obtained, after an overnight fast, by “tru-cut” needle (sample length/diameter = 20/1.2 mm) using a biopsy gun. At least one sample per patient was obtained. Lengths of liver specimens were recorded, as were the number of portal tracts. The sample was then assessed as being useful or not for histological diagnosis and fibrosis staging; samples of <10 mm length or <15 portal tracts were excluded. Biopsies were processed using standard procedures, formalin-fixed and paraffin-embedded. A single pathologist, who was blinded with respect to provenance of the samples, assessed the samples using haematoxylin-eosin, reticulin and Masson’s trichrome-stains to determine the grading and staging assignments according to Kleiner *et al.*^5^. This scoring system comprises four semi-quantitative features: steatosis, lobular inflammation, hepatocellular ballooning, and fibrosis. Steatohepatitis presence was not inferred from the NAS but, instead, was diagnosed taking into account patterns of histological distribution of lesions focusing on inflammatory activity and ballooning. Kleiner NAFLD Activity Score (NAS Score) and fibrosis stage were also calculated. NAS Score provides an overall score that comprises the degree of steatosis (score 0–3), lobular inflammation (score 0–3) and hepatocyte ballooning (score 0–2). *Hepatic steatosis* was quantified as the percentage of hepatocytes containing fat droplets, graded on a scale of 0–3 through subjective visual estimation of cells containing fat vacuoles. Steatosis grades were broadly categorised for severity: grade 0 or normal (up to 5% of hepatocytes affected); grade 1 or mild (5–33% of cells affected); grade 2 or moderate (33–66% showing steatosis); grade 3 or severe (>66% of hepatocytes showed fat storage). *Lobular inflammation* was assessed as: grade 0 (non-inflammation); grade 1 (<2 foci/x200 field); grade 2 (2–4 foci/x200 field); grade 3 (>4 foci/x200 field). *Ballooning* was evaluated as: stage 0 (none); stage 1 (a few balloon cells); stage 2 (many cells or prominent ballooning). *Fibrosis staging* was based on a 5-level scale: F0 = absence; F1 = perisinusoidal or periportal; F2 = perisinusoidal and portal/periportal; F3 = bridging fibrosis; F4 = cirrhosis. A further 2-level scale of fibrosis was applied: mild (F0-F1); advanced fibrosis (F2-F3-F4). Any adverse events from liver biopsy were reported.

### Magnetic resonance image acquisition

MR studies were conducted at the six University Hospitals using General Electric (Milwaukee, CT, USA) or Philips (Best, NL.) 1.5-Tesla whole-body systems within a period of six months from liver biopsy. Patients were examined in the supine position using a standard torso coil centred over the liver. No contrast medium was used, and the patient was encouraged to individual breath-holding capacity by the technologist. MRIs were sent to the referral Centre for processing in standard DICOM format. The images were processed and interpreted by two experienced engineers independently and, finally, a consensus was achieved. Both engineers were blinded to clinical and histopathological data (Table 1, Supplementary material). The entire liver was imaged and 6 sections were selected covering the whole organ. MR protocols for this study were performed in axial plane: SSFSE-T2 (Single Shot Fast Spin Echo T2-weighted), FAST-STIR (Fast Short inversion Time Inversion Recovery), inPHASE-outPHASE (in and out Phase) and DYNAMIC (See Table 1, Imaging Parameters, Supplementary material). DICOM files, field of view (FoV) and matrix sizes were configured specifically for each MR protocol; minimum and maximum window values were calculated so that each slice could be converted into a numerical matrix of pixels within the specific window range.

### MR imaging processing to define NASHMRI and FibroMRI imaging biomarkers

#### Development and standardisation of imaging biomarkers

Thirty-nine patients were consecutively included in the estimation cohort; 20 (51%) had steatohepatitis and 19 (49%) had significant fibrosis. The contour of the liver parenchyma is manually drawn in each slice. Each MR image is further divided according to a square grid that defines the set of samples (squares) to be processed. The spacing of the grid is chosen so that each sample square (from 10 × 10 pixels to 23 × 23 pixels, depending on image resolution and slice thickness) corresponds to an optimal volume of liver biopsy. Each sample is further analysed to exclude those containing artefacts, such as vessels or biliary ducts. In addition, those samples with >30% of its pixels outside the segmented area are discarded. Only those grid squares comprising liver parenchyma are analysed.

MR image features, segmentation algorithms, and implementation codes were developed in MATLAB (Matrix Laboratory, MathWorks, Natick, MA, USA) programming language. The software tool imports DICOM MR files and parses them, extracting all relevant information needed, including patient’s clinical and demographic data from the MR protocol.

The image-processing algorithms comprise the following steps (see image 2, Supplementary material):

The whole set of MR slices are presented to the user. The user, preferably those that contain the major liver section, must choose up to 6 consecutive slices.

In each selected image, the user outlines the liver boundary. When the parenchyma is segmented, a square grid is automatically over-layered. To achieve a sample size (of each square) equivalent to a volume of 15 to 24 mm^3^ of tissue, the quantity of pixels of each sample is computed using the FoV, the number of rows and columns of the image matrix and the slice thickness. Therefore, the final amount of samples processed varies for different MR sequences.

The software automatically discards those samples with >30% of the surface outside the segmentation line i.e. with a minimum of 70% pixels exhibiting liver parenchyma. The user must also reject samples that do not represent homogeneous liver tissue (i.e. those pixels containing vessels, ducts or other elements).

A total of 84 different mathematical image parameters or “estimators” are computed from each sample. The nature of these parameters ranges from simple statistical descriptors such as mean and standard deviation, to advanced image processing properties such as energy and entropy, geometrical properties like mean surface curvature, and spectral characteristics.

All calculated parameters for each sample (patient and protocol), are related to clinical features (biochemical parameters and histological scores) of NASH and fibrosis using logistic regression to determine the optimal combination of protocols and parameters.

#### Validation of imaging biomarkers

The imaging biomarkers that were developed were validated in a cohort of 87 patients. No differences were observed with respect to age, gender, steatosis degree, steatohepatitis or fibrosis distribution between the estimation and the validation cohorts (see Table 2). The average time consumed in MR studies was around 11 ± 3 minutes.

#### Comparison with biochemical biomarkers and transient elastography

NASH-MRI was compared with serum CK-18 levels. The FibroMRI was compared with Sydney Index, the NAFLD Fibrosis Score and the transient elastography.

### Statistical analyses of data

Software package SPSS 22.0 (SPSS, Chicago, IL, USA) was used to record data and to perform detailed statistical analysis. All p-values < 0.05 were considered statistically significant. Receiver operating characteristics (ROC) curves, which represent the trade-off between the true and false-positive rates, were used to differentiate the misclassified data between normal and disease status. The statistical method to compare the area under receiver operating characteristic (AUROC) curves was based on the method of Hanley *et al.* 48,49. NASH and significant fibrosis (F2-F4) were dichotomised as presence or absence of the feature. NASHMRI and FibroMRI were the outputs of the optical analyses and were defined as predictive models to detect steatohepatitis and significant fibrosis. Multiple logistic regressions were performed to define the final formula for NASH calculation (NASHMRI), and significant fibrosis (FibroMRI) presence. Sample size was intended to detect significant differences between histological diagnosis and NASHMRI and FibroMRI, using nQuery advisor v7.0 software. Sample size of the validation cohort was 84 patients with a significance level (alpha) of 0.05, 1 – power (beta) of 0.20, prevalence of steatohepatitis of 0.5 and, under the hypothesis of AUROC curve, a difference <0.12 (i.e. AUROC for NASHMRI of 0.83 and for histological steatohepatitis of 0.95).

## Additional Information

**How to cite this article**: Gallego-Durán, R. *et al.* Imaging biomarkers for steatohepatitis and fibrosis detection in non-alcoholic fatty liver disease. *Sci. Rep.* **6**, 31421; doi: 10.1038/srep31421 (2016).

## Supplementary Material

Supplementary Information

## Figures and Tables

**Table 1:** Baseline characteristics of the patient population: metabolic, demographic, and anthropometric data.

Parameter	Overall cohort (N = 126)
Age; y	51 ± 12
Male gender; %	78 (62%)
Body mass index; kg/m^2^	30.6 ± 4.8
Waist circumference; cm	102 ± 11
Caucasian ethnicity; %	100
Arterial hypertension; %	36.4
Diabetes; %	37.5
Cholesterol; mmol/L	8.5 ± 10.9
Triglycerides; mmol/L	5.9 ± 11.7
ALT; IU/L	73 ± 44
AST; IU/L	46 ± 39
GGT; IU/L	101 ± 101
Platelet count; ×10^9^	233 ± 57
Fasting glucose; mmol/L	5.6 ± 3.8
HOMA index	3.8 + 2.8
Insulin; mg/dL	14.9 ± 9.3
Albumin; g/dL	4.3 + 0.4
Sydney Index	0.31 + 0.31
NFS	−1.5 + 1.73
Transient elastography; kPa	7.6 + 6.1
CK-18; ng/ml	0.31 + 0.25
Liver biopsy length; mm	17.5 + 3.0

Values presented as mean ± SD, unless otherwise stated.

Footnotes Table 1: AST: Aspartate aminotransferase; ALT: Alanine aminotransferase; GGT: Gamma glutamyl transferase; NFS: NAFLD fibrosis score.

**Table 2:** Baseline characteristic comparisons between cohorts.

Parameter	Overall Cohort (N = 126)	Estimation Cohort (N = 39)	Validation Cohort (N = 87)	P
Age; y	51 ± 12	52 ± 11	50 ± 13	ns
Male gender	83 (66%)	29/39 (74%)	54 (62%)	ns
BMI; Kg/m^2^	30.6 ± 4.8	29.2 ± 4.8	31.1 ± 5.1	ns
Steatosis grade; %				ns
1	75 (60%)	21 (54%)	54 (62%)	ns
2	31 (24%)	10 (26%)	21 (24%)	ns
3	20 (15%)	8 (21%)	12 (14%)	ns
NASH; %	65 (51%)	21 (54%)	44 (51%)	ns
Fibrosis stage; %				ns
F0	52 (41%)	13 (33%)	39 (44%)	ns
F1	24 (19%)	7 (18%)	17 (20%)	ns
F2	27 (21%)	9 (23%)	18 (21%)	ns
F3	16 (13%)	7 (18%)	9 (10%)	ns
F4	7 (6%)	3 (8%)	4 (5%)	ns
