# Supplementary material for: Imaging biomarkers for steatohepatitis and fibrosis detection in non-alcoholic fatty liver disease
Source: Sci Rep. 2016 Aug 12;6:31421. doi: 10.1038/srep31421 (PMC4981860; doi:10.1038/srep31421)

**Imaging biomarkers for steatohepatitis and fibrosis detection in non-alcoholic fatty liver disease**

Rocío Gallego-Durán^1,2^, Pablo Cerro-Salido^3^, Emilio Gómez-González^3^, María Jesús Pareja^4^, Javier Ampuero^1,2^, María Carmen Rico^1,2^, Rafael Aznar^5^, Eduardo Vilar-Gomez^1,2^ , Elisabetta Bugianesi^6^, Javier Crespo^7^, Francisco José González-Sánchez^8^, Reyes Aparcero^9^, Inmaculada Moreno^10^, Susana Soto^11^, María Teresa Arias-Loste^7^, Javier Abad^12^, Isidora Ranchal^1,2^, Raúl Jesús Andrade^10^, Jose Luis Calleja^12^, Miguel Pastrana^13^, Oreste Lo Iacono^11^, Manuel Romero-Gómez^1,2,*^

^1^ UCM Digestive Diseases, Virgen Macarena-Virgen del Rocío University Hospitals, University of Sevilla, Sevilla, 41013, Spain

^2^ Instituto de Biomedicina de Sevilla (IBiS), University of Sevilla, Sevilla, 41013, Spain

^3^ Group of Interdisciplinary Physics, Engineering School, University of Sevilla, Sevilla, 41092, Spain

^4^ Pathology Unit, Valme University Hospital, University of Sevilla, Sevilla, 41014, Spain

^5^ Radiology Unit, Valme University Hospital, University of Sevilla, Sevilla, 41014, Spain

^6^ Medical Sciences, University of Torino, Torino, 10126, Italy

^7^ IDIVAL, Marqués de Valdecilla University Hospital, Gastroenterology and Hepatology Service, Santander, Spain

^8^ Radiology Unit, Marqués de Valdecilla University Hospital, Santander, 39008, Spain

^9^ UCM Digestive Diseases, Valme University Hospital, Sevilla, 41014, Spain

^10^ UCM Digestive Diseases and CIBERehd, Virgen de la Victoria University Hospital, Málaga, 29010, Spain

^11^ UCM Digestive Diseases, Tajo University Hospital, Madrid, 28300, Spain

^12^ UCM Digestive Diseases, Puerta de Hierro University Hospital, Madrid, 28222, Spain

^13^ Radiology Unit, Puerta de Hierro University Hospital, Madrid, 28222, Spain

*** Corresponding author**

**Contact information:**

Manuel Romero-Gómez, MD, PhD,

Unit for Clinical Management of Digestive Diseases,

Virgen Macarena-Virgen del Rocío University Hospitals,

University of Seville,

Avda. Manuel Siurot s/n,

41071 Sevilla,

Spain

Tel: (+34) 955 00 88 01

E-mail: [mromerogomez@us.es](mailto:mromerogomez@us.es)

**SUPPLEMENTARY MATERIAL**

**Supplementary Table 1**: Instructions to patients, preparation for imaging procedure, and imaging parameters recorded

| **INSTRUCTIONS** | - **Arms up if possible** - **Breath hold must be consistent** - **Supine position** - **Contrast: none** |
| --- | --- |

| **Imaging parameters** | **Sequence name** | | | |
| --- | --- | --- | --- | --- |
|  | SSFSE-T2 | FAST-STIR | In and out of PHASE | DYNAMIC |
| **Scan plane** | Axial | Axial | Axial | Axial |
| **Imaging options** | Breath hold | Breath hold | Breath hold | Breath hold |
| **Time (sec)** | 29.76 +10.05 | 340.98 + 104.10 | 38.10 + 6.66 | 35.44 + 90.33 |
| **FOV (mm)** | 450 | 400 | 410 | 375 |
| **Matrix size (px)** | 512x512 | 448x448 | 432x432 | 192x192 |

Sec: seconds; FoV: Field of View; mm: millimeters; px: pixel

**Supplementary Table 2**: Definition and properties of the estimators

| PROTOCOL | ESTIMATOR | NAME | DEFINITION AND PROPERTIES |
| --- | --- | --- | --- |
| **SSFSE-T2** | E3 | Harmonic mean | **The harmonic mean of a set of values, in this case the set of pixels of a sample, is a special type of media used when the average of rates is desired. The harmonic mean is the reciprocal of the arithmetic mean of the reciprocals** |
| **DYNAMIC** | E57 | Second order contrast | **Another texture attribute of a given sample, computed as a local grey level variation in the grey level co-occurrence matrix. It can be thought of as a linear dependency of grey levels of neighbouring pixels. If the neighbouring pixels are very similar in their grey level values then the contrast in the image is very low. In case of texture, the grey level variations show the variation of texture itself. High contrast values are expected for heavy textures and low for smooth, soft textures** |
| **FAST-STIR** | E73 | Weighted mean curvature | **Curvature is a feature used to describe image surface. The Weighted mean curvature is a value representing the mean of the curvatures mean at each pixel within the image sample.** |
| **SSFSE-T2** | E22 | Pearson’s asymmetry coefficient | **Pearson’s asymmetry coefficient, also called Pearson’s first coefficient of skewness, is a way to figure out the skewness of a distribution. It tells how far the distribution departs from symmetry.** |
| **DYNAMIC** | E6 | Mode | **The Mode of a set of numbers, in this case the pixel sample values, is the value that occurs most often.** |
| **DYNAMIC** | E31 | Column’s mean of multi-oriented co-occurrence matrix | **In image processing, co-occurrence matrices are used to analyse the texture of an image. A multi-oriented matrix computes the magnitude and orientation of the local gradient vector at each pixel position, so each pixel carries its grey value, its gradient magnitude and its gradient orientation.** |
| **DYNAMIC** | **E75** | **Maximum of main curvatures** | **In this case, the maximum value for all the primary curvatures (primary curvature from every pixel of the sample) is used as a surface attribute descriptor.** |

**Supplementary figure 1: Box plot of FibroMRI according to significant fibrosis detection.**

**
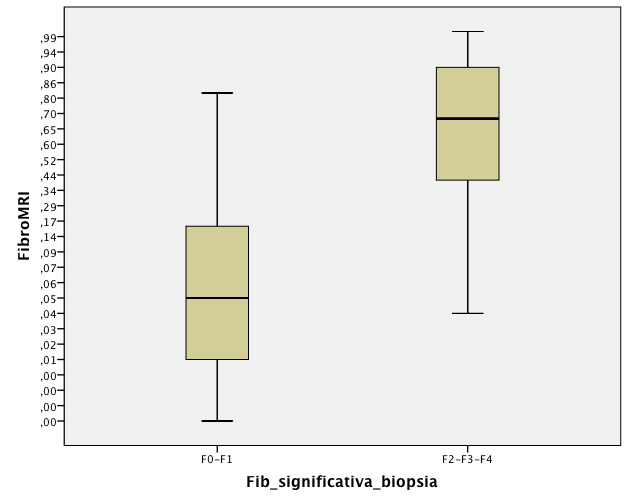
**

**Supplementary figure 2: Box plot of NASHMRI according to NASH presence.**

**
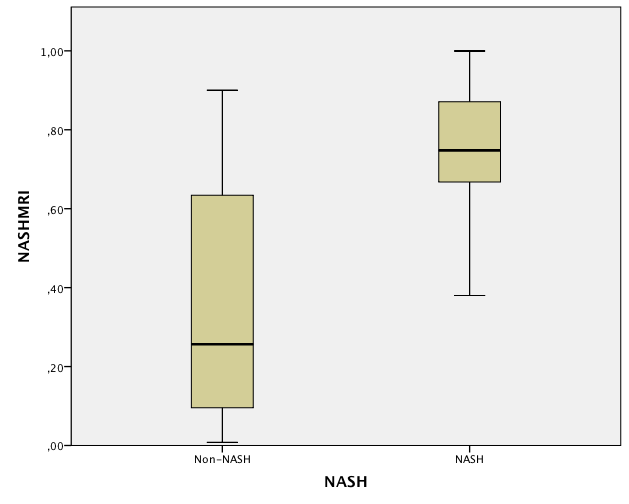
**

**Supplementary table 3:** STARD checklist for reporting of studies of diagnostic accuracy.

| **Section and Topic** | **Item**  **#** |  | **On page #** |
| --- | --- | --- | --- |
| TITLE/ABSTRACT/  KEYWORDS | 1 | Identify the article as a study of diagnostic accuracy (recommend MeSH heading 'sensitivity and specificity'). | 1 |
| INTRODUCTION | 2 | State the research questions or study aims, such as estimating diagnostic accuracy or comparing accuracy between tests or across participant groups. | 8 |
| METHODS |  |  |  |
| *Participants* | 3 | The study population: The inclusion and exclusion criteria, setting and locations where data were collected. | 8 |
|  | 4 | Participant recruitment: Was recruitment based on presenting symptoms, results from previous tests, or the fact that the participants had received the index tests or the reference standard? | 8 |
|  | 5 | Participant sampling: Was the study population a consecutive series of participants defined by the selection criteria in item 3 and 4? If not, specify how participants were further selected. | 8 |
|  | 6 | Data collection: Was data collection planned before the index test and reference standard were performed (prospective study) or after (retrospective study)? | 8 |
| *Test methods* | 7 | The reference standard and its rationale. | 8 |
|  | 8 | Technical specifications of material and methods involved including how and when measurements were taken, and/or cite references for index tests and reference standard. | 9-13 |
|  | 9 | Definition of and rationale for the units, cut-offs and/or categories of the results of the index tests and the reference standard. | 13 |
|  | 10 | The number, training and expertise of the persons executing and reading the index tests and the reference standard. | 10 |
|  | 11 | Whether or not the readers of the index tests and reference standard were blind (masked) to the results of the other test and describe any other clinical information available to the readers. | 11 |
| *Statistical methods* | 12 | Methods for calculating or comparing measures of diagnostic accuracy, and the statistical methods used to quantify uncertainty (e.g. 95% confidence intervals). | 13 |
|  | 13 | Methods for calculating test reproducibility, if done. | 13 |
| RESULTS |  |  |  |
| *Participants* | 14 | When study was performed, including beginning and end dates of recruitment. | 8 |
|  | 15 | Clinical and demographic characteristics of the study population (at least information on age, gender, spectrum of presenting symptoms). | Table 1 |
|  | 16 | The number of participants satisfying the criteria for inclusion who did or did not undergo the index tests and/or the reference standard; describe why participants failed to undergo either test (a flow diagram is strongly recommended). | 14 |
| *Test results* | 17 | Time-interval between the index tests and the reference standard, and any treatment administered in between. | 9 |
|  | 18 | Distribution of severity of disease (define criteria) in those with the target condition; other diagnoses in participants without the target condition. | Table 2 |
|  | 19 | A cross tabulation of the results of the index tests (including indeterminate and missing results) by the results of the reference standard; for continuous results, the distribution of the test results by the results of the reference standard. | Figures |
|  | 20 | Any adverse events from performing the index tests or the reference standard. | 10 |
| *Estimates* | 21 | Estimates of diagnostic accuracy and measures of statistical uncertainty (e.g. 95% confidence intervals). | 14 |
|  | 22 | How indeterminate results, missing data and outliers of the index tests were handled. | 15 |
|  | 23 | Estimates of variability of diagnostic accuracy between subgroups of participants, readers or centers, if done. | 15 |
|  | 24 | Estimates of test reproducibility, if done. | 15 |
| DISCUSSION | 25 | Discuss the clinical applicability of the study findings. | 16-20 |

**IMAGE PROCESSING STEPS TO CALCULATE NASHMRI**

Supplementary Figure 3: MR images acquisition


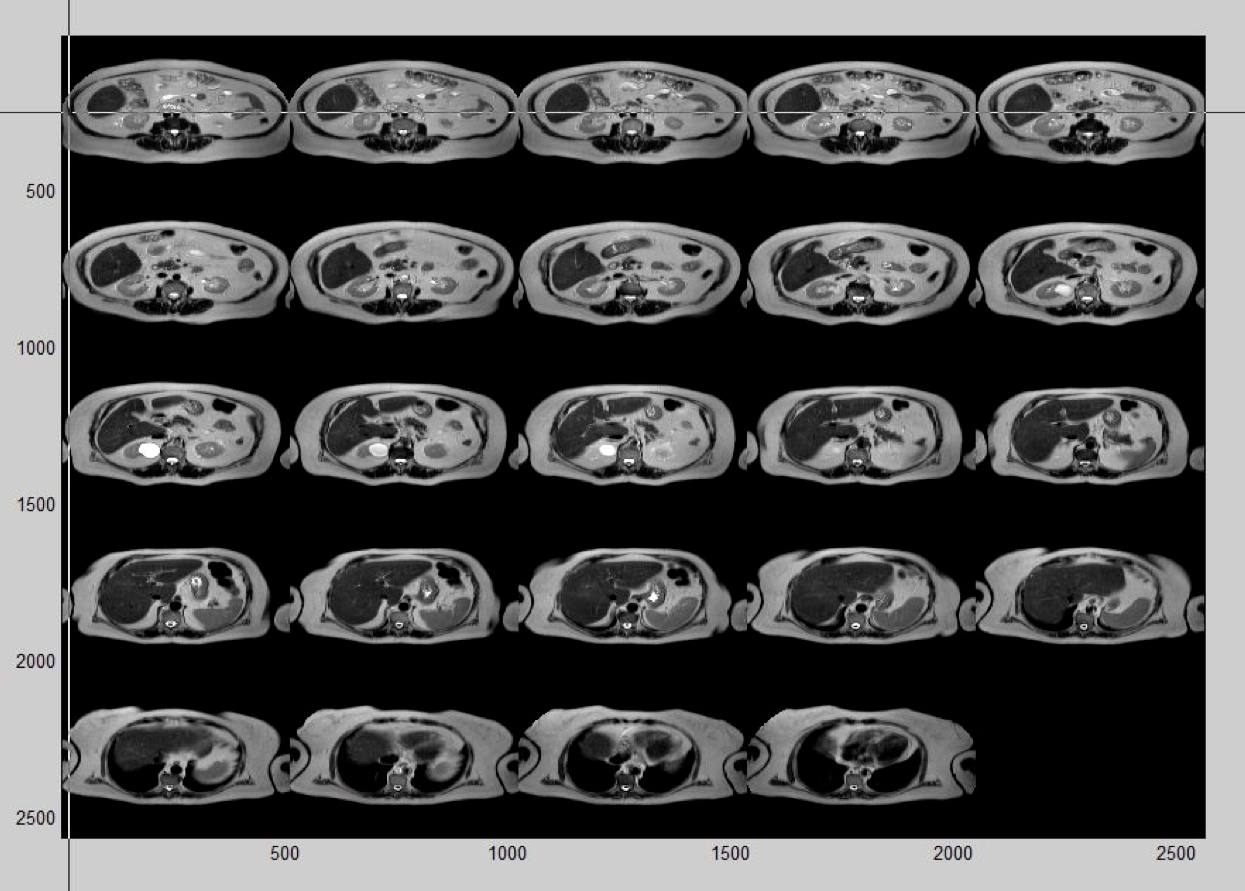


Supplementary Figure 4: Selection of six MR images containing liver


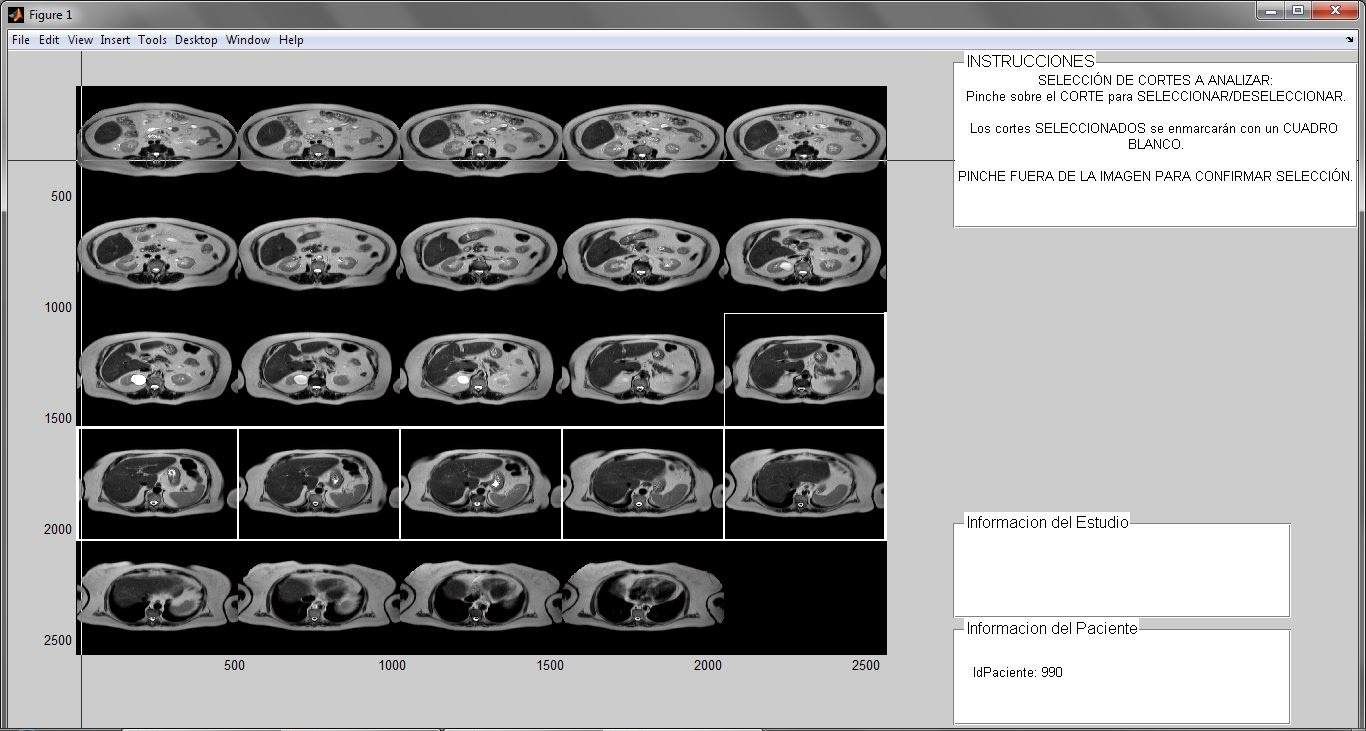


Supplementary Figure 5: Presentation of selected slice


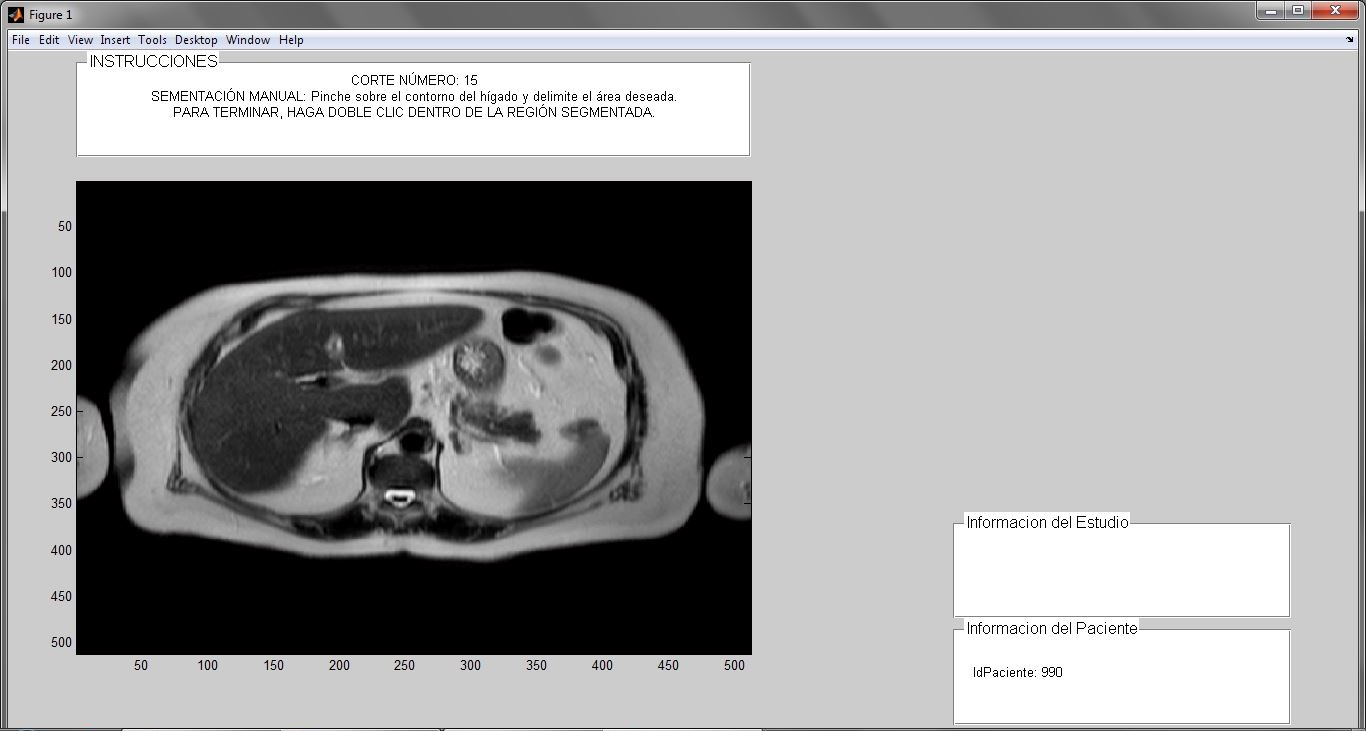


Supplementary Figure 6: Manual outlining of liver boundaries


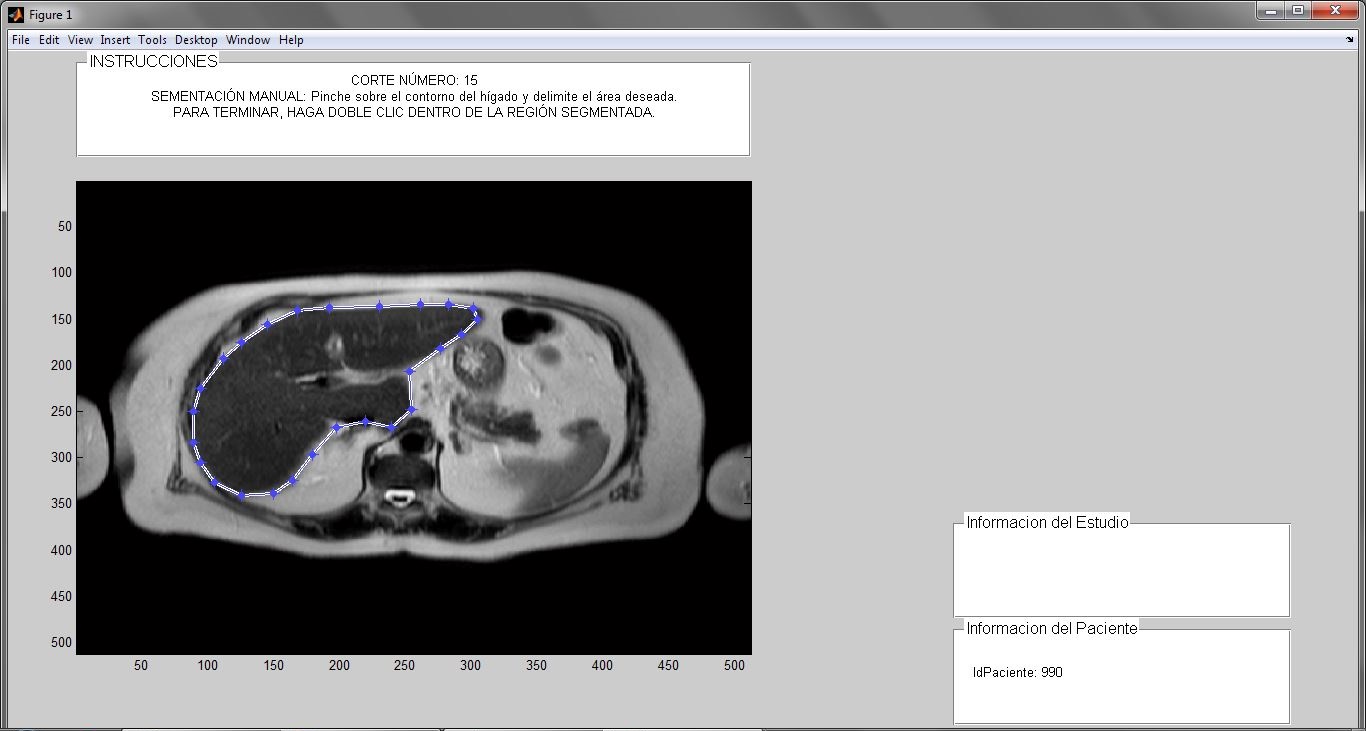


Supplementary Figure 7: Segmentation and overlapping of the square grid


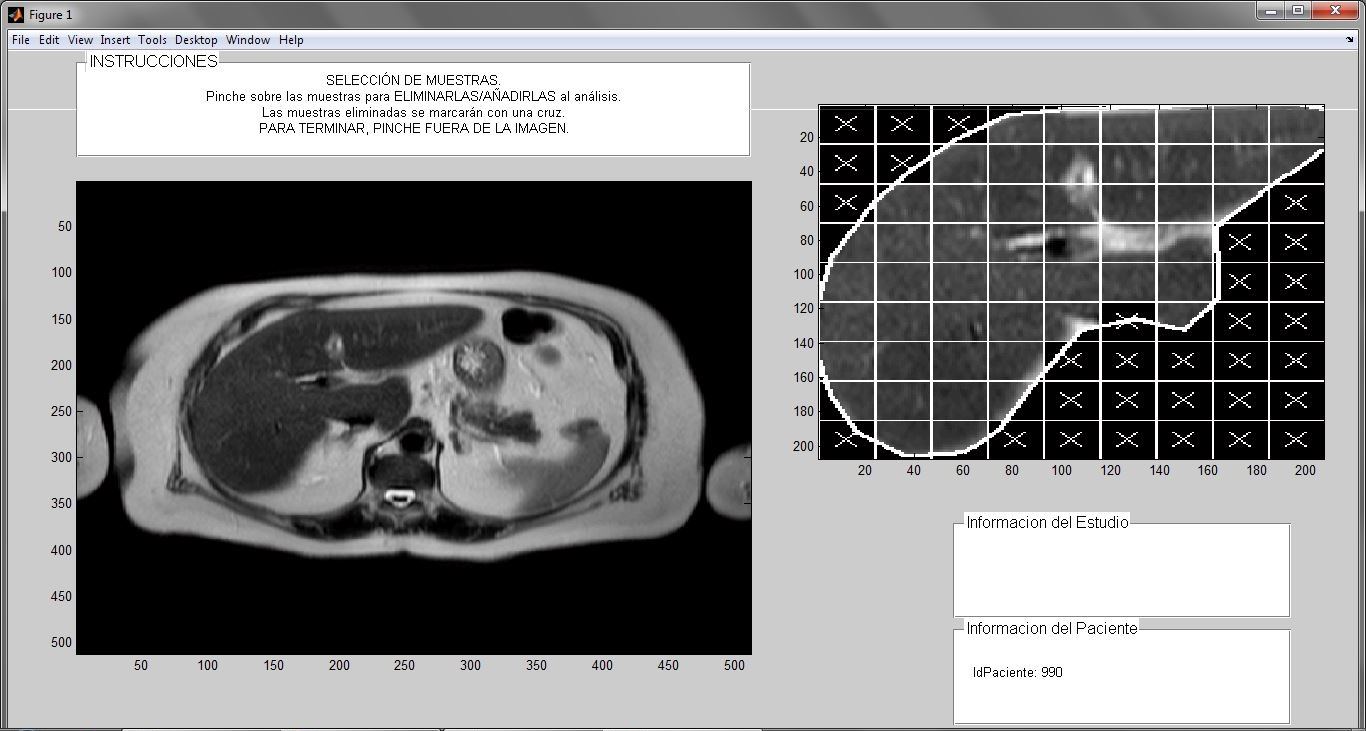


Supplementary Figure 8: Valid sample selection process


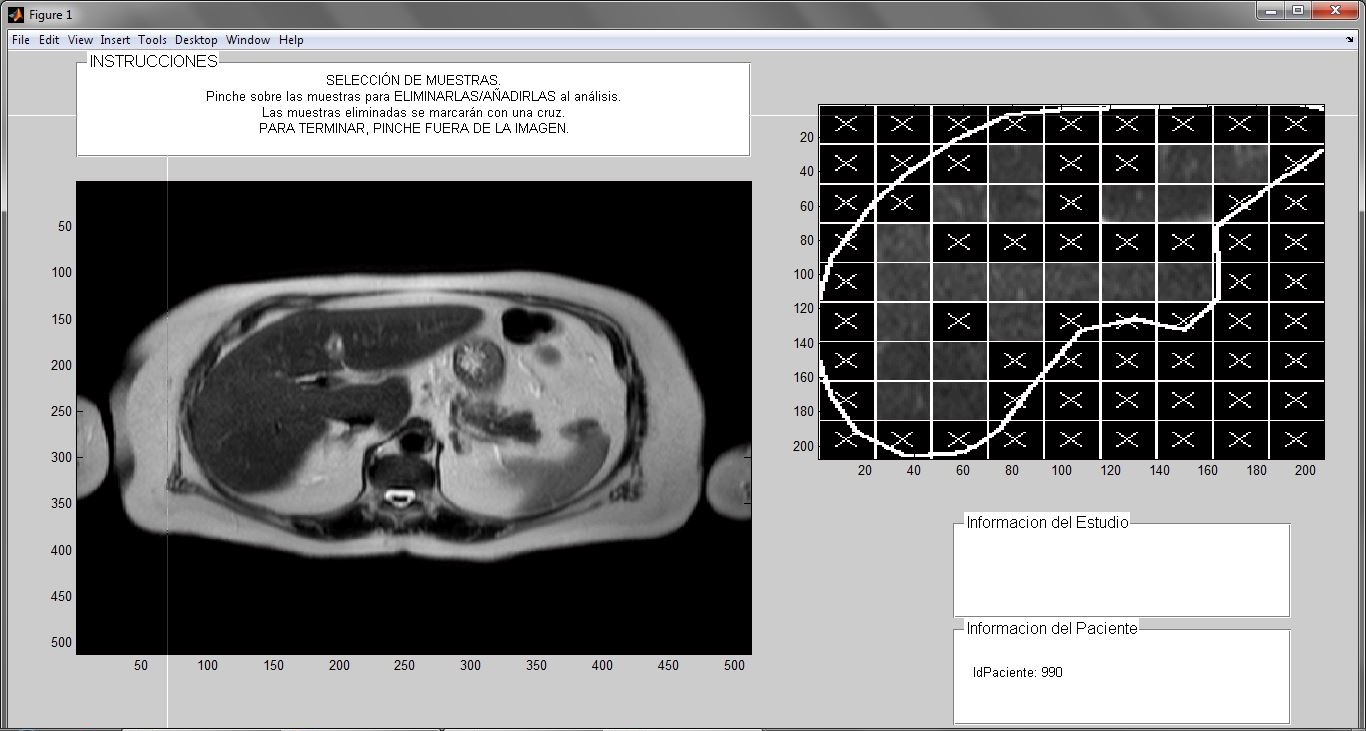


Supplementary Figure 8: Database analysis, calculation and interpretation of NASHMRI


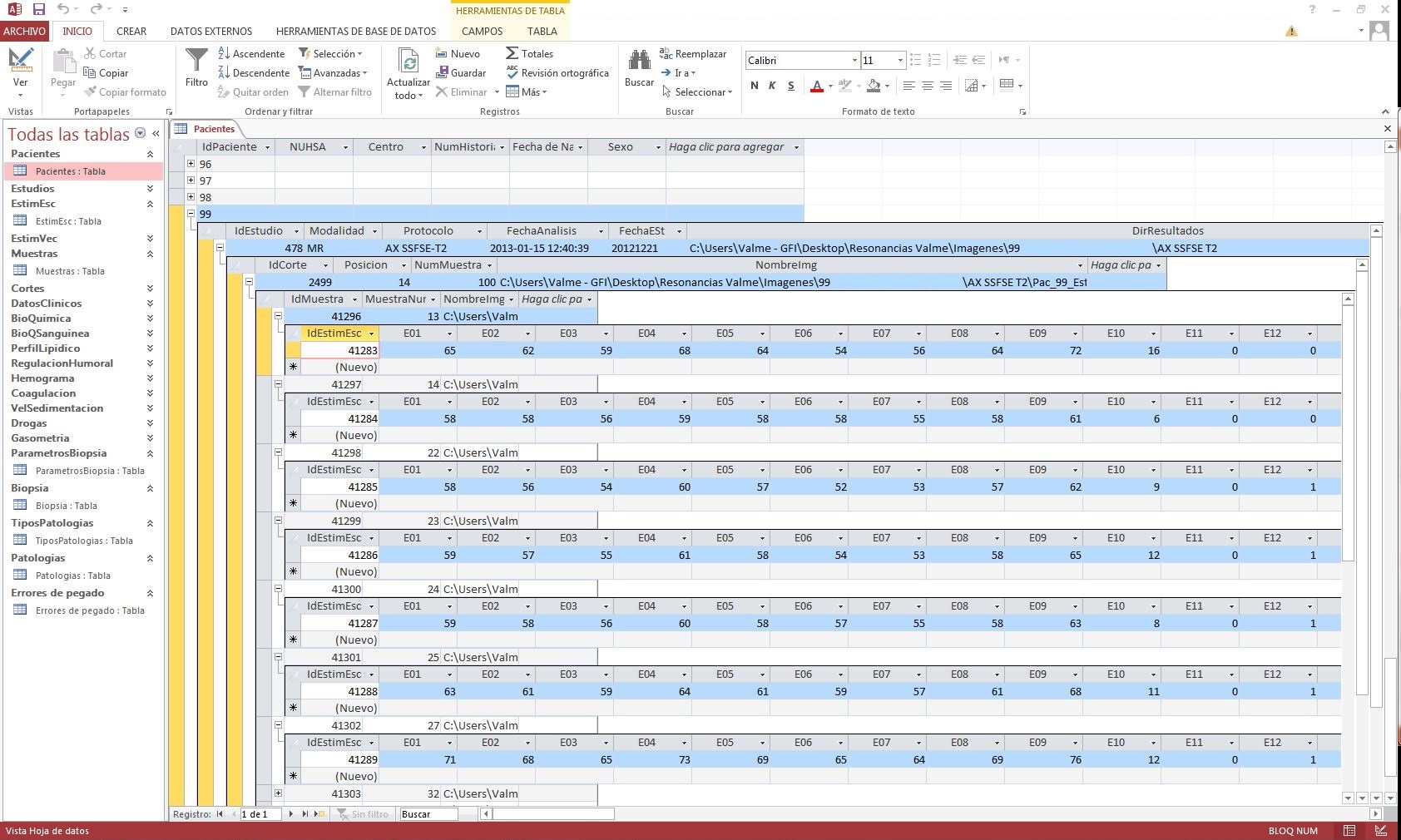


NASHMRI = 1/ 1 + e 1.654 - 0.079*E3(SSFSE-T2) – 0.127*E57(DYNAMIC)*E73(FAST-STIR)


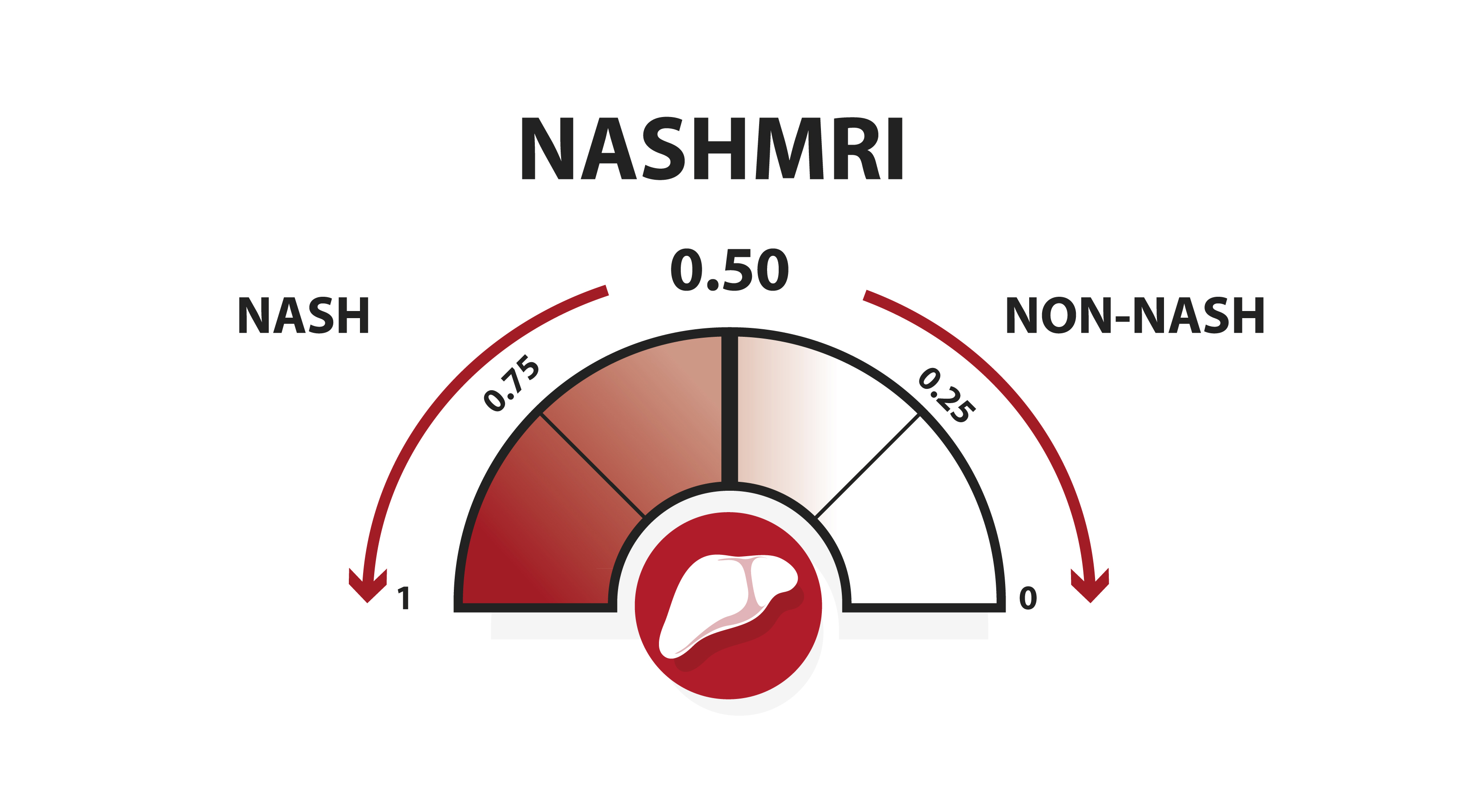

Supplement: Supplementary Information [file srep31421-s1.doc]
